# *ABCB1 C1236T*, *G2677TA* and *C3435T* Genetic Polymorphisms and Antidepressant Response Phenotypes: Results from a Portuguese Major Depressive Disorder Cohort

**DOI:** 10.3390/ijms25105112

**Published:** 2024-05-08

**Authors:** Marlene Santos, Luis Lima, Serafim Carvalho, Andreia Brandão, Fátima Barroso, Agostinho Cruz, Rui Medeiros

**Affiliations:** 1 REQUIMTE/LAQV, Escola Superior de Saúde, Instituto Politécnico do Porto, Rua Dr. António Bernardino de Almeida, 400, 4200-072 Porto, Portugal; mes@ess.ipp.pt (M.S.); agostinhocruz@ess.ipp.pt (A.C.); 2 Molecular Oncology & Viral Pathology, IPO-Porto Research Center (CI-IPOP), Portuguese Institute of Oncology, 4200-072 Porto, Portugal; 3 Experimental Pathology and Therapeutics Group, IPO-Porto Research Center (CI-IPOP), Portuguese Institute of Oncology, 4200-072 Porto, Portugal; luis.carlos.lima@ipoporto.min-saude.pt; 4 Hospital de Magalhães Lemos, Centro Hospitalar Universitário de Santo António, 4149-003 Porto, Portugal; smicarval@gmail.com; 5 Instituto Universitário de Ciências da Saúde, 4585-116 Gandra, Portugal; 6 Cancer Genetics Group, IPO-Porto Research Center (CI-IPOP), Portuguese Oncology Institute of Porto (IPO-Porto), 4200-072 Porto, Portugal; 7 REQUIMTE/LAQV, Instituto Superior de Engenharia do Porto, Instituto Politécnico do Porto, Rua Dr. António Bernardino de Almeida 431, 4249-015 Porto, Portugal; mfb@isep.ipp.pt; 8 Research Department, Portuguese League Against Cancer (NRNorte), 4200-172 Porto, Portugal

**Keywords:** *ABCB1*, polymorphisms, antidepressant phenotypes

## Abstract

P-glycoprotein (P-GP) is a transporter molecule expressed on the apical surface of capillary endothelial cells of the Blood–Brain Barrier (BBB), whose activity heavily influences drug distribution, including antidepressants. This transporter is encoded by *ABCB1* gene, and genetic variations within *ABCB1* gene have been proposed to affect drug efflux and have been previously associated with depression. In this context, we aimed to evaluate the role of *C1236T*, *G2677TA* and *C3435T ABCB1* genetic polymorphisms in antidepressant treatment phenotypes from a cohort of patients harboring Major Depressive Disorder. Patients enrolled in the study consisted of 80 individuals with Major Depressive Disorder, who took part in a 27-month follow-up study at HML, Portugal. To investigate the correlation between *ABCB1* polymorphisms and antidepressant response phenotypes, DNA was extracted from peripheral blood, and *C1236T*, *C3435T* and *G2677TA* polymorphisms were genotyped with TaqMan^®^ SNP Genotyping Assays. Despite the fact that the evaluated polymorphisms (*C1236T*, *C3435T* and *G2677TA*) were not associated with treatment resistant depression, or relapse, we observed that patients carrying TT genotype of the *C3435T* polymorphism remit earlier than the ones carrying CC or CT genotypes (10.2 weeks vs. 14.9 and 21.3, respectively, *p* = 0.028, Log-rank test). Since we found an association with *C3435T* and time to remission, and not to the absence of remission, we suggest that this polymorphism could have an impact on antidepressant drug distribution, and thus influence on the time to remission will occur, without influencing the risk of remission itself.

## 1. Introduction

Plasmatic drug levels are influenced by several pharmacokinetic molecules, namely transporter proteins and metabolizing enzymes. P-glycoprotein (P-GP) is a transporter molecule, whose activity heavily influences drug distribution [1]. This efflux pump is encoded by *ABCB1* gene and is principally expressed in the Blood–Brain Barrier (BBB), lower gastrointestinal tract, liver, placenta and CD56 lymphocytes, influencing drug bioavailability [1]. P-GP is found to influence intestinal drug absorption and, in some cases, limit oral drug bioavailability. At the luminal surface of endothelial cells of BBB and placenta, this molecule is known to protect the brain and fetus from xenobiotics [2].

Some evidence suggests that antidepressants such as amitriptyline, trimipramine, venlafaxine, doxepin, citalopram, sertraline and paroxetine are all substrates of the P-GP transporter [3]. In contrast, fluoxetine and mirtazapine, two commonly prescribed antidepressants, are not substrates of P-GP [3]. *ABCB1* knockout mice present altered pharmacokinetics as a result of many drugs, specifically the accumulation in the brain, liver and gall bladder of P-GP substrate drugs [4]. For instance, concentration of paroxetine in the brain was higher in knockout than in wild-type mice, suggesting that the lack of P-GP activity is associated with elevated antidepressant levels in the brain [4].

Also, this transporter function is thought to be inhibited by some drugs, for example verapamil [5]. In a mice model, inhibition P-GP by verapamil enhanced the intracerebral concentration of imipramine, supporting the hypothesis that P-GP activity restricts brain levels of certain antidepressants, including imipramine [6].

**Figure 1:**
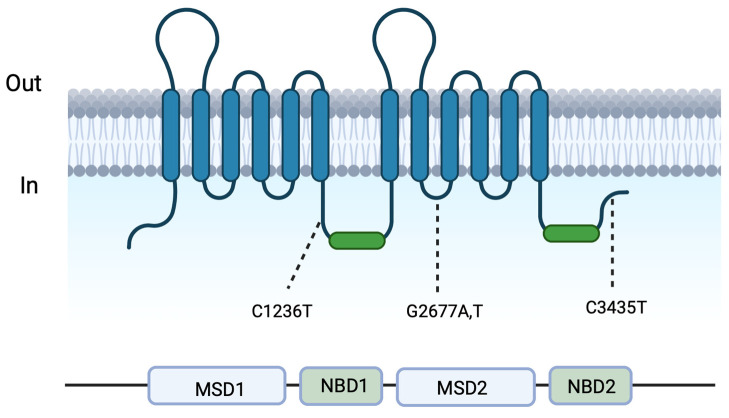
The figure illustrates the two-dimensional structure of *ABCB1*, with positions representing polymorphisms in the *ABCB1* gene. MSDs: membrane-spanning domains. NBDs: nucleotide (ATP)-binding domains. Created with BioRender.com.

The *ABCB1* gene is located in chromosomal region 7q21, and includes 28 exons and encodes a 1280-amino acid transporter [3]. Genetic variants among *ABCB1* gene *C1236T*, *C3435T* and *G2677TA* (Figure 1) may have a direct influence in function and expression of P-GP, and are likely to influence BBB and intestinal drug transport [7]. Regarding well-known *C3435T* polymorphisms, this is correlated with an altered expression in the intestines, altering the bioavailability of P-GP substrate drugs: T-allele is associated with low intestinal expression, while C-allele is associated with increased P-GP [8]. 

Several papers have addressed the influence of functional genetic polymorphisms in *ABCB1* gene on the response to antidepressant drugs (AD). Usually, these papers evaluated response in the usual window of 4–8 weeks after initiating an antidepressant drug in a clinical trial setting. The first paper to evaluate the effect of *ABCB1* polymorphisms in remission was conducted by Uhr et al. [7]. Based on his findings, the *ABCB1* genotyping was applied as a diagnostic tool in the Max-Plank Institute. Following their study, several investigators tried to replicate these findings, with contradictory results. While some studies [9,10,11,12,13] validated the findings of Uhr et al., others failed to show an association of *ABCB1* genetic polymorphisms with AD response [14,15,16,17,18]. A recent meta-analysis that investigated the association between six *ABCB1* single-nucleotide polymorphisms (rs1045642, rs2032582, rs1128503, rs2032583, rs2235015 and rs2235040) and antidepressant treatment outcomes only found a significant association between rs1128503 and treatment response [19]. The polymorphisms under evaluation were not associated with treatment remission and tolerability [19].

PharmGKB clinical annotations provide information about variant-drug pairs based primarily on variant annotations and incorporating variant-specific prescribing guidance. The level of evidence of the possible impact of *ABCB1* genetic variants in antidepressant drugs efficacy or toxicity can be observed in Table 1, and of other drugs in Supplementary data. According to the PharmGKB database [20], there are several *ABCB1* variants, including C1236T, *C3435T* and *G2677TA*, that impact antidepressants efficacy and/or toxicity, and all of them are level 3. Level 3 evidence means that the association may be based on a single study annotated in PharmGKB, or there may be several studies that failed to replicate the association [20].

Taking in consideration the contradictory results published in the literature, and the fact that we previously reported that *ABCB1* polymorphisms were associated with the risk of depression among male patients [21], in this paper we wanted to address the influence of *ABCB1* polymorphisms in AD response phenotypes, in a cohort of Major Depressive Disorder (MDD) Portuguese patients.

## 2. Results

### 2.1. Association between Sociodemographic and Clinical Characteristics and ABCB1 Genotypes

In this study, the associations between sociodemographic and clinical characteristics and *ABCB1* genotypes (*C1236T*, *C3435T* and *G2677TA* polymorphisms) were analyzed (Table 2). The investigation revealed no statistically significant differences in the mean age of participants across the genotypes for the *C1236T* (*p* = 0.807), *C3435T* (*p* = 0.246) and *G2677TA* polymorphisms (*p* = 0.867). Furthermore, the frequency of previous depressive episodes did not significantly vary among the genotypes for all polymorphisms analyzed, indicating no significant correlation between these genetic variations and the historical incidence of depressive episodes.

The analysis of time to remission showed variability across genotypes for the *C1236T* and *C3435T* polymorphisms; however, only the *C3435T* polymorphism demonstrated a statistically significant difference (*p* = 0.027), suggesting its potential influence on remission latency in depression treatment. In contrast, time to relapse and total duration of study participation did not show significant differences across genotypes for the C1236T, *C3435T* and *G2677TA* polymorphisms. Additionally, baseline and final Beck Depression Inventory (BDI) scores, along with the change in BDI scores across the genotypes, were evaluated. The results indicated no significant differences for the *C1236T* (*p* = 0.276 for baseline BDI, *p* = 0.535 for final BDI), *C3435T* (*p* = 0.265 for baseline BDI, *p* = 0.497 for final BDI) and *G2677TA* polymorphisms (*p* = 0.647 for baseline BDI, *p* = 0.762 for final BDI), suggesting that these specific *ABCB1* genetic variations do not significantly impact the severity or change in depressive symptoms throughout the study.

### 2.2. Genotype Frequencies of ABCB1 Polymorphisms and Treatment Outcome

The genotype distribution of *ABCB1* polymorphisms were evaluated among the different treatment response phenotypes (Table 3). No significant deviations from the Hardy–Weinberg equilibrium proportions were observed for either of the SNPs (*p* > 0.05).

For the *C1236T* polymorphism (rs1128503), the data indicated no significant correlation between the genotype (whether CC, CT, TT or T carriers) and the incidence of relapse, as reflected by the *p*-values that spanned from 0.579 to 0.793. This trend persisted when examining the relationship between these genotypes and TRD, with no genotype variation demonstrating a statistically significant association, underscored by *p*-values ranging from 0.698 to 0.810. The odds ratios did not point to any substantial risk alteration tied to the T allele when compared to the CC reference group.

Similarly, the *C3435T* polymorphism (rs1045642) showed no notable linkage to either clinical outcome. The analyses for relapse and TRD across genotypes (CC, CT, TT and T carriers) yielded *p*-values that consistently indicated a lack of significant association, ranging from 0.535 for relapse to 1.000 for treatment resistance. The observed odds ratios further supported the conclusion that no meaningful differences in risk of relapse or treatment resistance exist among the genotypes.

The results for the *G2677TA* polymorphisms (rs2032582) aligned with the patterns observed in the *C1236T* and *C3435T* polymorphisms. No significant associations were discovered between the various genotypes and relapse, with *p*-values extending to 1.000 in some cases, suggesting no visible impact of genotype on relapse rates. The evaluation of treatment resistance exhibited an increased, but not statistically significant, odds ratio of 1.610 (*p* = 0.391), indicating a lack of compelling evidence to suggest a significant relationship between the *G2677TA* genotypes and TRD. Notably, the analysis was constrained by limited data for some genotypes (e.g., GA and TA), denoted by **, which implies data limitations or very small sample sizes. Even when data was stratified according to gender, none of the evaluated polymorphisms (*C1236T*, *C3435T* and *G2677TA*) were associated with TRD or relapse (*p*-values ranged from 0.091 to 0.975). 

**Figure 2:**
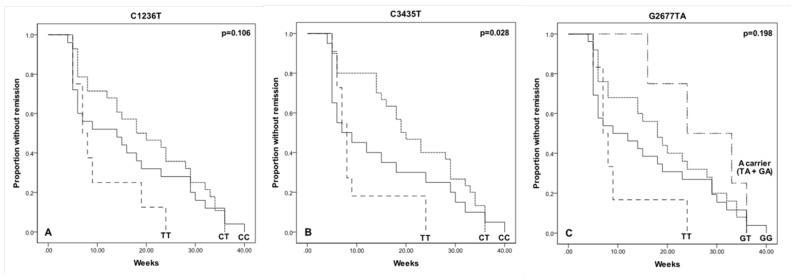
Effect of *ABCB1* genotypes on time to remission in MDD patients. Kaplan–Meier curves depict the impact of *ABCB1* genotypes (*C1236T*, *C3435T*, *G2677TA*/ (**A**), (**B**) and (**C**) respectively) on remission timing in MDD patients, analyzed via Log-rank test. Significant difference was observed only for the *C3435T* genotype (*p* = 0.028), indicating its potential influence on time to remission.

Given the initial association observed between the *C3435T* polymorphism and time to remission as presented in Table 2, we undertook a more detailed investigation of this relationship. Specifically, patients who harbor TT genotype were found to experience a significantly shorter time to remission compared to those with either the CC or CT genotypes. The mean time to remission for TT genotype carriers was approximately 10.2 weeks, which stands in contrast to the longer durations observed in patients with the CC and CT genotypes, averaging 14.9 and 21.3 weeks, respectively. This difference reached statistical significance with a *p*-value of 0.028, as determined by the Log-rank test, and is visually depicted in Figure 2. Univariate Cox Regression analysis revealed that patients presenting TT genotype had an increased probability of remission (HR = 2.187; 95% CI: 1.088–4.396; *p* = 0.028). These findings suggest that the TT genotype may confer a faster response to depression treatment, highlighting a potential genetic influence on the efficacy of therapeutic interventions. Kaplan–Meier analysis showed no differences regarding time to remission for the other polymorphisms under evaluation (Figure 2), nor the time to relapse for each genotype of the three evaluated polymorphisms (Log-rank test; *p* > 0.05).

### 2.3. Evaluating ABCB1 Polymorphisms as Expression Quantitative Trait Loci

We used the publicly available data from Open Targets Genetics (https://genetics.opentargets.org/ accessed on 20 March 2024) to investigate whether the three polymorphisms, *C1236T*, *C3435T* and *G2677TA*, act as expression quantitative trait loci (eQTLs), modulating the expression of *ABCB1*, and potentially other additional genes. Our analysis revealed that *C1236T* polymorphisms and eQTLs are for modulating the expression of four genes, namely *RUNDC3B* and *ABCB1*, and to a lesser extent *CROT* and STEAP4 genes. More specifically, this polymorphism decreases the expression levels of *RUNDC3B* and *ABCB1* in heart atrial appendage and in blood and testis, respectively. In contrast, according to the eQTL catalog, *C1236T* increases the expression levels of *CROT* and *STEAP4* in blood. The *C3435T* polymorphism (rs1045642) is also an eQTL for the *ABCB1*, *RUNDC3B* and *ABCB4* genes. Similarly to the *C1236T* polymorphism, *C3435T* decreases the expression levels of *RUNDC3B* and *ABCB1* in heart atrial appendage and in blood and testis, respectively. Interestingly, this SNP lowers the expression levels of *ABCB4* on blood but increases its levels on artery tibial tissue. Regarding the *G2677TA* trialelic polymorphism, which is dependant on the transition, only allele G is eQTL for reducing the expression of *ABCB1* on testis and *RUNDC3B* on atrial appendage.

## 3. Discussion

In this study, we evaluated the association of *ABCB1* genetic polymorphisms and antidepressant treatment response phenotypes in a cohort of Portuguese MDD patients. Despite *ABCB1* being one of the most studied genes among antidepressants pharmacogenomics, the literature results are contradictory. Here, we observed that *C3435T* and *ABCB1* genetic polymorphisms impact time to remission, where we showed that patients carrying TT genotype of the *C3435T* polymorphism remit earlier than the ones carrying CC or CT genotypes.

The results observed herein suggest that the evaluated *ABCB1* polymorphisms were not associated with relapse or TRD development. Moreover, when we extended our analysis to examine the time to relapse across the various genotypes of the evaluated polymorphisms, the Kaplan–Meier survival analysis did not reveal any significant differences. This part of the study aimed to understand whether the genetic variations under investigation could also predict the longevity of remission, thereby affecting the likelihood of relapse post-treatment. Despite the clear impact on time to remission, our findings indicate that the genotypes of the *C3435T* polymorphism, as well as the other evaluated polymorphisms, do not significantly alter the course of recovery in terms of relapse duration. This suggests that while certain genetic markers may influence the speed of initial treatment response, they do not necessarily predict the stability of that response over time.

**Figure 3:**
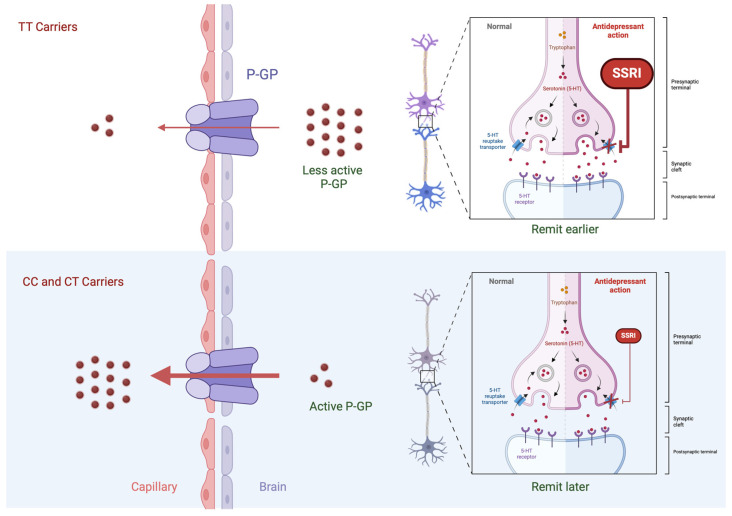
Possible impact of the *C3435T* genetic polymorphism on antidepressant efflux. 5-HT: serotonin; SSRI: Selective Serotonin Reuptake Inhibitor; P-GP: P-glycoprotein. Arrow displays the antidepressant’s efflux from P-glycoprotein. Created with BioRender.com.

Regarding the *C1236T* (rs1128503), the lack of association we observed was in accordance with several reports, including the study of Kato et al. [13], Uhr et al. [7], Singh et al. [10], as well as the GENEDEP study [14]. On the opposite, the study of Dong et al., showed an association of this polymorphism and antidepressant remission in patients treated with Fluoxetine [22]. Concerning the *G2677TA* polymorphism, we detected that this polymorphism was not associated with the studied AD response phenotypes. Our observations were in accordance with the reports from Laika et al. [23], Gex-fabry et al. [11], Mihaljevic Peles et al. [24], Uhr et al. [7] and also the GENEDEP study [14]. Contrarily, the studies of Kato et al. [13] and Nikisch et al. [25], and the subgroup of patients treated with Venlafaxine in the Singh study [10], reported a significant association with this genetic polymorphism and antidepressant response. Additionally, regarding the *C3435T* polymorphism, only the studies of Singh et al. (the venlafaxine group) and Lin et al. [12] corroborated an association between this polymorphism and treatment response. However, most of the studies reported lack of association [7,11,13,18,24,25]. The only study conducted with a Portuguese population, with a cross-sectional study design, found that carriers of the TTT-haplotype had a higher likelihood to be remitters compared with the non-TTT and TTT–TTT haplotypes [26]. A meta-analysis, published in 2015, which reviews the effect of *ABCB1* genetic polymorphisms and antidepressant outcome, reported that no association was observed between treatment outcome and any of the SNPs explored in this study [27]. This result remained even when data were stratified for ethnicities, hospitalization status and co-medication. However, time to remission was not evaluated in the meta-analysis. Taking into consideration that we found an association with *C3435T* and time to remission, and not to the absence of remission (TRD phenotype), we can hypothesize that this polymorphism could have an impact in antidepressant delivery, and thus influence the moment remission will occur, without influencing the risk of remission itself. Since we found that *C3435T* polymorphism (rs1045642) is an eQTL for *ABCB1*, affecting its expression, we might expect that TT carriers, who may accumulate higher levels of the drug in their brain, would remit quicker than non-carriers, as seen in our results. A putative mechanism for this is explained in Figure 3. This theory is supported by the results of Singh [10], whose study found out that patients carrying TT of the *C3435T* polymorphism required a significantly lower dose of 11 mg of escitalopram to remit, while TC and CC carriers demanded a superior dose of 24 and 19 mg, respectively. According to this study, the lower P-GP efflux in TT carriers may have greater Central Nervous System (CNS) bioavailability of antidepressant drugs at the lower doses reached during this 8-week study, and hence a significantly greater remission rate. Conversely, these findings appear to support the hypothesis that C carriers of the *C3435T* may have higher ABCB1 efflux and reduced central nervous system bioavailability of the antidepressants studied [10], explaining why these patients display longer times to remission (Figure 3). In fact, in a study from Xu & Wang (2022) [28], a significant correlation of *C3435T* polymorphisms with *ABCB1* DNA methylation status was found [29]. Their results have proven that the methylation degree of the *ABCB1* promoter region in different genotypes of *C3435T* was significantly different. However, this was not the case of *C1236T* and G2677T/A genetic polymorphisms [29]. Furthermore, it is known that some drugs, including antidepressants, such as tricyclic antidepressants (e.g., amitriptyline, clomipramine, desipramine, and SSRI (citalopram, fluoxetine, paroxetine and sertraline) may inhibit ABCB1 [1]. However, the mechanism for this effect has not been explained, but can be related to modified gene expression regulatory mechanisms, for instance methy-lation status. Interestingly, a recent review revealed that antidepressants can influence BDNF methylation status [28].

The primary limitation of our study stems from its sample size. Despite this constraint, to our knowledge, our research is pioneering in evaluating *ABCB1* genotypes alongside TRD and relapse phenotypes, time to remission and time to relapse within the Portuguese population undergoing antidepressant treatment. It stands distinct from the sole other study in this domain, which involved 79 patients. That study focused on remission and adverse reaction outcomes within a cross-sectional framework, differing from our approach [26]. Furthermore, and although our data suggest that an accumulation of higher antidepressant levels in the brain may account for a faster of remission, this theory needs to be demonstrated directly to support this relationship. Additionally, and considering the nature of the therapeutic algorithm applied, different drugs were used in each step of the algorithm, so we could not stratify our results, based on the information about the P-GP substrate categories. However, it is likely that this issue did not largely influence our results since the work of Magalhães et al. only evaluated fluoxetine and observed a higher likelihood to be remitters among TTT haplotype [26].

Given the highlighted limitations, in this study we corroborated the findings reported in most of the published reports, which were the lack of association of these three polymorphisms and TRD, relapse and time to relapse, but observed that *C3435T* influences time to remission in our cohort. Further studies should include this parameter when evaluating antidepressants response.

## 4. Materials and Methods

### 4.1. Patients

Patients participating in this study comprised 80 individuals diagnosed with MDD, who engaged in a 27-month follow-up investigation at Hospital Magalhães Lemos (HML), as described previously [30]. This cohort included 21 males and 59 females, aged from 18 to 60 years, with a median age of 41.5 years old (mean age 40.48; standard deviation 11.06). The Structured Clinical Interview for DSM Axis I Disorders (SCID-I) was employed to evaluate major depression, while Axis II Disorders were assessed for personality disorders using the SCID-II. Depression severity was gauged through the Beck Depression Inventory (BDI), with an entry criterion set at a BDI score of 20. Exclusions from the study were substance abuse, personality disorders, psychiatric diseases with non-depressive psychotic symptoms, severe physical sickness and more than one past depressive episode. The Texas Medication Algorithm Project (TMAP) for MDD treatment algorithm guided the selection of optimal medication strategies [31]. The initial medication selection followed the TMAP algorithm’s first-line recommendations. Pharmacotherapeutic effectiveness was assessed based on changes in the BDI score, after a minimum of 6 weeks at appropriate doses as per the TMAP protocol. Adherence to the TMAP protocol allowed for modifications in antidepressant medication if the therapeutic response was insufficient or intolerable.

### 4.2. Antidepressant Phenotypes

Remission was defined as achieving a BDI score below 10 after six weeks of receiving at least one suitable antidepressant, coupled with no longer meeting SCID-I criteria for Major Depressive Disorder. Relapse was characterized as any depressive episode occurring post-remission during the follow-up period. Treatment-resistant depression was identified when a patient failed to attain a BDI score below 10 and continued to meet SCID-I criteria for MDD after undergoing at least two adequate antidepressant treatments with different drugs within the current episode [32].

### 4.3. Sample Collection and DNA Extraction

Peripheral blood samples were obtained using the standard venipuncture method and collected in tubes containing EDTA. Genomic DNA was then isolated from these whole blood samples using the E.Z.N.A. Blood DNA Kit (Omega Bio-tek), following the protocol provided by the manufacturer. Subsequently, the extracted DNA was stored at −20 °C for preservation.

### 4.4. C1236T, G2677TA, C3435T ABCB1 Polymorphism Analysis

*ABCB1* polymorphisms were selected based on their putative or proven functionality in previews reports, a minor allele frequency of 15% and possible correlation with antidepressant response. TaqMan^®^ SNP Genotyping Assays C___7586662_10; C_11711720C_30 and C_11711720D_40; C___7586657_20 were used to evaluate C1236T, G2677A/T, *C3435T* and *ABCB1* genetic polymorphism analysis, respectively (Applied Biosystems, Foster City, CA, USA), as we previously described [18]. Briefly, we used an Applied Biosystems 7300 Real Time PCR System to perform the reactions (Applied Biosystems, Foster City, CA, USA) which contained in a 5 μL final volume mixture: 1× TaqMan Genotyping Master Mix (Applied Biosystems, Foster City, CA, USA), 900 nM of each primer, 200 nM of probes labeled with either FAM or VIC and 10 ng of extracted DNA. The thermal cycling conditions were: 10 min at 95 °C followed by 45 cycles (or 50 cycles for G2677A/T) of 15 s at 95 °C and 1 min at 60 °C. The ABI PRISM^®^ 7300 Sequence Detection System Allelic discrimination was used to perform the measurement of endpoint fluorescence (Applied Biosystems, Foster City, CA, USA). Genotyping data was read blind to the clinical course of illness and, in case of ambiguous genotypic data, experiments were repeated for determining the genotype.

### 4.5. Statistical Analysis

The statistical analysis was performed with IBM^®^SPSS^®^ (ver.22.0) and Epi Info (ver.6.04a). Chi-square (χ2) analysis was used to compare the categorical variables with a 5% level of significance. The association between genotypes and the risk of developing the evaluated phenotypes was determined by Odds ratio (OR) with a 95% confidence interval (CI). For tables containing cells with less than five individuals, Fisher’s Exact Test was used. Correlation between genotypes and time to relapse and time to remission was accomplished with Kaplan–Meier survival curves, which were compared by Log-rank statistical test. Univariate Cox Regression analysis was used to assess the effect of *ABCB1* genotypes on time to remission.

### 4.6. eQTL Analysis

Expression quantitative trait loci (eQTLs) can affect gene expression by various mechanisms, including altering promoter activity. To evaluate whether the *C1236T*, *C3435T* and *G2677TA* polymorphisms are eQTLs, we used the publicly available eQTL Catalogue from Open Targets Genetics [33]. This eQTL Catalogue aggregates data from eQTL Catalogue [34], QTLGen [34] and GTEx [35].

### 4.7. Ethical Approval

This study was approved by the Hospital Magalhães Lemos ethics board. Furthermore, each participant provided written informed permission in accordance with “The Code of Ethics of the International Medical Association” (Declaration of Helsinki) after being briefed on the study’s purpose and methods.

## 5. Conclusions

In this study, we explored the influence of *ABCB1* polymorphisms on depression treatment outcomes, focusing on TRD, relapse, time to remission and time to relapse. Our findings revealed that *C1236T* and *G2677TA* do not influence antidepressant phenotypes in our cohort of Portuguese patients treated with antidepressants. However, the *C3435T* polymorphism showed a statistically significant impact on time to remission, suggesting its potential role in modifying the latency of remission in depression treatment. Specifically, individuals carrying the TT genotype experienced a lower time to remission compared to those with CC or CT genotypes, supporting the hypothesis that C carriers of the *C3435T* polymorphism may experience higher P-GP efflux, resulting in reduced CNS bioavailability of antidepressants. This mechanism provides a plausible explanation for the observed longer times to remission among these patients, highlighting the critical role of *ABCB1* in antidepressant treatment. Furthers studied are required to confirm the results provided herein and to in vitro evaluate the impact of this polymorphism in the antidepressants efflux. Additionally, it would be very interesting to assess if antidepressants may modify *ABCB1* gene regulatory mechanisms (for instance, DNA methylation) affecting its expression, as well as influence the clinical course of the disease. 

## Figures and Tables

**Table 1:** PharmGKB clinical annotations about *ABCB1* genetic variants and antidepressants.

PharmGKB ID	Level	Variant	*Gene*	Drugs	Phenotype Categories
1183619292	3	rs10248420	*ABCB1*	amitriptyline; citalopram; paroxetine; venlafaxine	Efficacy
1183619303	3	rs10280101	*ABCB1*	amitriptyline; citalopram; paroxetine; venlafaxine	Efficacy
655384846	3	rs1045642	*ABCB1*	Nortriptyline	Toxicity
1447943597	3	rs1045642	*ABCB1*	Venlafaxine	Efficacy
1447943591	3	rs1045642	*ABCB1*	Agomelatine	Efficacy
1447943608	3	rs1045642	*ABCB1*	Selective serotonin reuptake inhibitors	Efficacy
1452426500	3	rs1128503	*ABCB1*	Antidepressants	Efficacy
1183619312	3	rs11983225	*ABCB1*	amitriptyline; citalopram; paroxetine; venlafaxine	Efficacy
1183619317	3	rs12720067	*ABCB1*	amitriptyline; citalopram; paroxetine; venlafaxine	Efficacy
1444703230	3	rs2032582	*ABCB1*	Fluoxetine	Efficacy
982032743	3	rs2032582	*ABCB1*	clomipramine; lithium; nefazodone; paroxetine; venlafaxine	Toxicity
619523414	3	rs2032583	*ABCB1*	amitriptyline; antidepressants; citalopram; fluvoxamine; paroxetine; sertraline; venlafaxine	Efficacy
619523422	3	rs2235015	*ABCB1*	amitriptyline; antidepressants; citalopram; paroxetine; venlafaxine	Efficacy
1183619322	3	rs2235040	*ABCB1*	amitriptyline; citalopram; paroxetine; venlafaxine	Efficacy
1183629315	3	rs2235067	*ABCB1*	amitriptyline; citalopram; paroxetine; venlafaxine	Efficacy
1183689585	3	rs28401781	*ABCB1*	citalopram; fluoxetine; paroxetine; sertraline	Efficacy
1183629305	3	rs4148739	*ABCB1*	amitriptyline; citalopram; fluoxetine; paroxetine; sertraline; venlafaxine	Efficacy
1183629310	3	rs4148740	*ABCB1*	amitriptyline; citalopram; paroxetine; venlafaxine	Efficacy
982031026	3	rs7787082	*ABCB1*	amitriptyline; citalopram; paroxetine; venlafaxine	Efficacy

**Table 2:** Sociodemographic and clinical characteristics according to the genotype.

Variables	*ABCB1 C1236T* Polymorphism				*ABCB1 C3435T* Polymorphism				*ABCB1 G2677TA* Polymorphism					
	CC	CT	TT	*p* Value	CC	CT	TT	*p* Value	GG	GT	GA	TT	TA	*p* Value
**Age (mean and SD)**	40.6 (10.5)	41.0 (11.4)	38.5 (12.3)	0.807	43.0 (10.7)	40.1 (11.5)	37.1 (10.0)	0.246	41.3 (10.7)	40.2 (11.6)	49.0	38.0 (10.5)	38.3 (15.9)	0.867
**Sex, female (%)**	18 (56.2)	31 (83.8)	10 (90.9)	0.013	14 (53.2)	30 (76.9)	15 (100)	0.004	18 (56.3)	30 (83.3)	0 (0)	8 (100)	3 (100)	0.009
**Number of previous depressive episodes**	1.3 (0.5)	1.5 (0.5)	1.5 (0.5)	0.299	1.4 (0.5)	1.5 (0.5)	1.5 (0.5)	0.719	1.3 (0.5)	1.6 (0.5)	1.0	1.5 (0.5)	1.3 (0.6)	0.400
**Time to remission (weeks), mean (SD)**	16.2 (12.1)	20.0 (11.3)	10.5 (7.1)	0.102	14.9 (12.3)	21.3 (10.9)	10.2 (6.9)	0.027 ^#^	15.6 (12.1)	18.9 (11.0)	16.0	10.0 (7.0)	31.0 (6.2)	0.096
**Time to relapse (weeks) mean (SD)**	30.6 (13.8)	45.7 (25.2)	19.7 (17.9)	0.177	34.8 (12.1)	31.7 (16.9)	37.0 (37.6)	0.930	30.6 (13.8)	44.9 (23.1)	ND	9.5 (4.9)	ND	0.077
**Total time in the study (weeks) mean (SD)**	59.7 (30.5)	67.3 (28.4)	54.3 (33.1)	0.359	34.6 (6.8)	26.1 (4.2)	30.1 (7.8)	0.312	62.8 (32.6)	64.8 (28.5)	95.0	47.6 (28.2)	59.9 (5.6)	0.497
**Baseline BDI score, mean (SD)**	29.2 (7.6)	27.4 (6.8)	25.3 (8.4)	0.276	29.5 (7.9)	27.6 (6.94)	25.6 (7.4)	0.265	28.8 (7.6)	27.9 (7.2)	24.0	24.5 (8.9)	26.3 (2.5)	0.647
**Final BDI score, mean (SD)**	16.1 (14.3)	13.0 (10.6)	13.6 (13.2)	0.535	16.6 (15.2)	12.9 (12.7)	14.4 (12.5)	0.497	15.8 (14.6)	13.9 (10.6)	1.0	12.1 (13.1)	16.0 (12.2)	0.762
**BDI change, mean (SD)**	12.9 (11.1)	14.4 (11.1)	11.6 (8.7)	0.720	12.8 (11.5)	14.7 (10.4)	11.0 (10.3)	0.501	13.0 (11.2)	14.0 (11.1)	23.0	12.4 (8.4)	10.3 (10.4)	0.872

*p* value: ANOVA test; ^#^ Independent-samples Kruskal-Wallis test.

**Table 3:** Genotype frequencies of *ABCB1* polymorphisms under investigation and outcomes.

		Relapsed							Resistant (TRD)						
		No		Yes		OR	CI 95%	*p*-Value	No		Yes		OR	CI 95%	*p*-Value
		N	%	N	%				N	%	N	%			
***C1236T*** **(rs1128503)**	CC	18	40.0	7	43.8	1.0	Referent	-	25	41.0	7	36.8	1.0	Referent	-
	CT	22	48.9	6	37.5	0.701	[0.200–2.462]	0.579	28	45.9	9	47.4	1.148	[0.373–3.537]	0.810
	TT	5	11.1	3	18.7	1.543	[0.288–8.250]	0.673 *	8	13.1	3	15.8	1.339	[0.279–6.434]	0.698 *
	T carrier	27	60.0	9	56.3	0.857	[0.270–2.717]	0.793	36	59.0	12	63.2	1.190	[0.411–3.445]	0.748
***C3435T*** **(rs1045642)**	CC	13	29.5	5	33.3	1.0	Referent	-	18	30.5	5	27.8	1.0	Referent	-
	CT	24	54.6	6	40.0	0.650	[0.166–2.546]	0.535	30	50.9	9	50.0	1.080	[0.313–3.731]	0.903
	TT	7	15.9	4	26.7	1.486	[0.299–7.389]	0.694 *	11	18.6	4	22.2	1.309	[0.288–5.948]	1.000 *
	T carrier	31	70.5	10	66.7	0.839	[0.239–2.938]	0.783	41	69.5	13	72.2	1.141	[0.354–3.681]	0.825
***G2677TA*** **(rs2032582)**	GG	19	42.2	7	43.8	1.0	Referent	-	26	42.6	6	31.6	1.0	Referent	-
	GT	18	40.0	7	43.8	1.056	[0.308–3.612]	0.931	25	41.0	11	57.9	1.907	[0.612–5.939]	0.262
	GA	1	2.2	0	0	**	**	1.000 *	1	1.6	0	0.0	**	**	1.000 *
	TT	4	8.9	2	12.5	1.357	[0.202–9.126]	1.000 *	6	9.8	2	10.5	1.444	[0.232–9.005]	0.650 *
	AA	0	0.0	0	0.0	-	-	-	0	0.0	0	0.0	-	-	-
	TA	3	6.7	0	0.0	**	**	0.557 *	3	4.9	0	0.0	**	**	1.000 *
	Variant carrier	26	57.8	9	56.3	0.940	[0.297–2.971]	0.915	35	57.4	13	68.4	1.610	[0.540–4.798]	0.391

TRD: Treatment Resistant Depression. OR: odds ratio.CI: confidence interval. * Fisher exact test. ** One cell count is 0; unable to calculate OR.

## Data Availability

The datasets generated during the current study are available from the corresponding author upon reasonable request.

## Supplementary Materials

The following supporting information can be downloaded at: https://www.mdpi.com/article/10.3390/ijms25105112/s1.
